# 

**Published:** 1888-06

**Authors:** 


CONTENTS: JUNE, 1888, VOL. III., NO. 12.

ORIGINAL COMMUNICATIONS.—Pelvic Peritonitis—By W. C. Fisher, M. D., Galveston .......... 504
Is the World Satisfied with the Medical Profession?—By L. W. Cocke, M. D., San Marcos .......... 508
A Prevalent Affection much Overlooked—By E. Meierhof, M. D., New York .......... 513
Paroxysmal Idiopathic Abdominal Pulsations—By A. H. McCord, M. D., Rusk .......... 519
Was it Cocaine? .......... 520
CORRESPONDENCE.—The Loco Plant; Letter from Dr. B. F. Kingsley, San Antonio .......... 522
A Letter from Our Arkansas Correspondent; Congenital Occlusion of the Anus .......... 524
CULLINGS FROM CONTEMPORARIES.—Edited by T. J. Bennett; Items .......... 525
EDITORIAL.—The Texas Medical College and Hospital .......... 530
Close of Vol. III .......... 533
MEDICAL NEWS AND MISCELLANY.—Marriages, Deaths, Removals, etc .......... 534
ADVERTISERS' NOTICES .......... 535
GENERAL INDEX ..........

